# Food security and marine capture fisheries: characteristics, trends, drivers and future perspectives

**DOI:** 10.1098/rstb.2010.0171

**Published:** 2010-09-27

**Authors:** Serge M. Garcia, Andrew A. Rosenberg

**Affiliations:** 1 FAO Fisheries and Aquaculture Management Division, Rome, Italy; 2 Conservation International, University of New Hampshire, Durham, NH, USA

**Keywords:** capture fisheries, food security, future, overfishing, environmental degradation, conservation

## Abstract

World population is expected to grow from the present 6.8 billion people to about 9 billion by 2050. The growing need for nutritious and healthy food will increase the demand for fisheries products from marine sources, whose productivity is already highly stressed by excessive fishing pressure, growing organic pollution, toxic contamination, coastal degradation and climate change. Looking towards 2050, the question is how fisheries governance, and the national and international policy and legal frameworks within which it is nested, will ensure a sustainable harvest, maintain biodiversity and ecosystem functions, and adapt to climate change. This paper looks at global fisheries production, the state of resources, contribution to food security and governance. It describes the main changes affecting the sector, including geographical expansion, fishing capacity-building, natural variability, environmental degradation and climate change. It identifies drivers and future challenges, while suggesting how new science, policies and interventions could best address those challenges.

## 1. Introduction

According to the United Nations Department of Economic and Social Affairs (UN-DESA 2009), the world population is expected to grow from the present 6.8 billion people to about 9 billion by 2050, mostly in developing countries (5.6–7.9 billion). With a growing world population and recurrent problems of hunger and malnutrition plaguing many communities, e.g. in South Asia and Sub-Saharan Africa, food security is of major societal and international concern. Fishery resources are an important source of proteins, vitamins and micronutrients, particularly for many low-income populations in rural areas, and their sustainable use for future global food security has garnered significant public policy attention. In the context of variable and changing ecosystems, and despite some progress, the challenges of maintaining or restoring fisheries sustainability and stock sizes, reducing environmental impact and degradation, and improving local and global food security remain immense.

Marine capture fisheries are a critical component of this picture. Their production is close to the maximum ecosystem productivity (NRC 2006), cannot be increased substantially in the future and could decline if not properly managed, leaving the world to solve a significant new food deficit. The 2002 World Summit on Sustainable Development (WSSD) called on States to ‘maintain or restore stocks to levels that can produce the maximum sustainable yield with the aim of achieving these goals for depleted stocks on an urgent basis and, where possible, not later than 2015’. The world is far from meeting this target, and this paper addresses the underlying issues and considers the future implications.

## 2. Selected characteristics of the fishery system

### (a) Production

**Figure 1.**
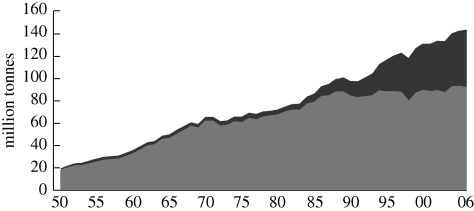
World capture and aquaculture production. Black, China; grey, world excluding China. Source: FAO (2009).

Current global fisheries production has been increasing since records commenced, except during the two World Wars (figure 1).

**Figure 2.**
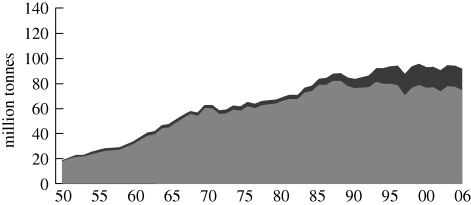
World capture fisheries production. Dark grey, China; light grey, world excluding China. Source: FAO (2009).

According to The Food and Agriculture Organization of the United Nations (FAO 2009, p. 3), fisheries produced close to 144 million tonnes of fish (live weight equivalent) in 2006, of which 82 million tonnes were from marine capture fisheries (figure 2), 10 million tonnes from inland capture fisheries, 32 million tonnes from inland aquaculture and 20 million tonnes from marine aquaculture. Aquaculture grew faster than any other food-producing sector and if sustained, will continue to augment capture fisheries production in response to global demand, supplying more than 50 per cent of aquatic food consumption by 2015 (Bostock *et al*. 2010). High seas catches have increased from below 2 million tonnes in 1950 to more than 10 million tonnes (FAO 2009, p. 14) and the taxonomy of the 133 species caught indicates growing deep-water fishing with reported catch close to 4 million tonnes. Altogether and despite reporting uncertainties, the world catch of marine capture fisheries may well have reached the upper limit of 100 million tonnes proposed by Gulland (1971). The ceiling for inland capture fisheries is highly uncertain, although there is some indication that additional growth is possible (FAO 2009, p. 8; Welcomme *et al*. 2010).

In addition to this, Illegal, Unreported and Unregulated (IUU) fishing is the major source of undocumented catches (FAO 2001). Agnew *et al*. (2009) estimated present IUU catches at 11–26 million tonnes, worth 10–20 billion USD annually. Information on IUU is increasing as societal concern grows, and as international and national governance mechanisms strengthen. Nonetheless, trends are not known, and the uncertainty in the estimates is substantial.

Discarding of unwanted catch in 1990–2000 has been estimated by FAO at 9.5 million tonnes (Kelleher 2005) or about 10 per cent of reported landings. Some studies have indicated that discarding rates may be substantially greater, regionally or globally (Harrington *et al*. 2005; Davies *et al*. 2009), but more recent estimates are not available. Discards appear to have decreased from about 27 million tonnes in 1980–1990 (Alverson *et al*. 1994) owing to bycatch reduction efforts as well as an increasing use of bycatch for local consumption, aquaculture feeds, etc.

### (b) Resources and environmental issues

The fact that the ceiling in marine fisheries production has been reached is illustrated by the state of marine resources. Relative to the level that would support maximum sustainable yield, 20 per cent of targeted fishery resources are moderately exploited, 52 per cent are fully exploited with no further increases anticipated, 19 per cent are overexploited, 8 per cent are depleted and 1 per cent are recovering from previous depletion (FAO 2009, p. 7). Similar figures have been compiled for US and Canada domestic fisheries, although a recent study of 10 well-studied ecosystems revealed five in which fishing pressure is declining owing to increased management (Worm *et al*. 2009). However, in European Community waters, more than 80 per cent of stocks are overexploited or depleted (European Commission 2007).

**Figure 3.**
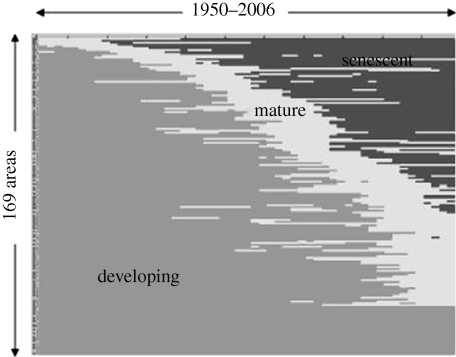
Chronology of resource development phases in 169 national fishing areas (1950–2006). Source: Garcia (2009*a*,*b*).

The first overview study of the state of marine fisheries resources by country (Garcia 2009*a*,*b*), using FAO statistics for 1950–2006, confirms that globally, the maximum average level of bottom fish and small pelagic fish production has been reached within the last decade. Catches of crustaceans and cephalopods are still growing, perhaps owing to reduced stocks of their predators but also owing to increased targeting because of their high price. At national or sub-national level, the analysis showed that during the last decade, 30 per cent of fishing areas were still ‘growing’ (increasing production), 30 per cent were ‘mature’ (stagnating production) and 40 per cent were ‘senescent’ (decreasing production, some of which for many decades; figure 3).

**Figure 4.**
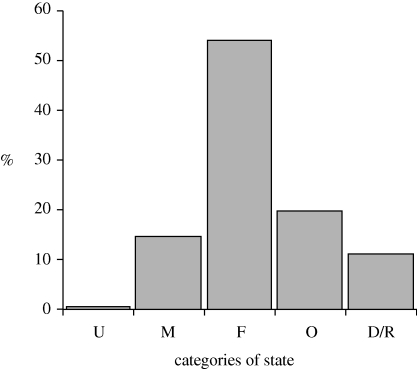
Distribution of annual landings (average 1998–2002) by category of resource state in FAO terminology. U, undeveloped; M, moderately developed; F, fully developed; O, overfished; D/R, depleted and recovering. Data source: FAO (2009).

It is also important to weigh the state of stocks by their importance in terms of maximum potential. The data are not available to fully explore this relationship but table 1 and figure 4, concerning about 75 per cent of recent landings (average 1998–2002), indicate that, respectively, 14.1 per cent of world production (about 11 million tonnes), 57.3 per cent (about 41 million tonnes) and 28.6 per cent (about 22 million tonnes) come from stocks considered, respectively, as underexploited or moderately exploited, fully exploited and overexploited, and depleted or recovering.

**Table 1.** Landings (average 1998–2002) by category of state of resources. U, undeveloped; M, moderately developed; F, fully developed; O, overfished; D/R: depleted and recovering. Landings data source: FAO (2005).

		categories					
	total	U	M	F	O	D/R	unknown
total (1000 tonnes)	75 828	440	7485	32 384	10 404	5767	19 348
total (%)	100	0.6	9.9	42.7	13.7	7.6	25.5
total known (%)	100	0.8	13.3	57.3	18.4	10.2	
total known (%)	100	14.1		57.3	28.6

While producing food, employment, livelihood and wealth, fisheries can also generate a significant level of environmental impact on target and non-target resources as well as on sensitive habitats (NRC 2002; Lokkeborg 2005; FAO 2008). Marine debris from lost fishing gear can continue to fish and entangle a wide variety of wildlife. Destructive fishing and IUU fishing aggravate impacts of fishing on the food web and can alter ecosystem structure and function, and ultimately productivity and resilience to the impacts of other drivers such as climate change. This large and crucial subject cannot be dealt with here in any level of detail but it is clear that without serious efforts to define and reduce such impacts, marine ecosystems will risk much greater negative pressure, and policy conflicts between conservation and fisheries could reduce the scope to develop sustainable and productive fisheries.

### (c) Fishing capacity and sectoral diversity

The structural and functional diversity of the sector needs to be carefully considered when considering its trends and future scenarios. Relevant typological dimensions include:

— scale of technology and investment separating small-scale and large-scale fisheries;
— business organization, ranging from artisanal (family business) to industrial (corporate);
— objectives such as: production of food for self-consumption (subsistence fisheries), fish meal and oils (reduction fisheries); the supply of local or international markets (commercial fisheries); or recreation (sport/recreational fisheries);
— target resources: e.g. high-value bottom fish fisheries or low-value small pelagic fisheries;
— type of jurisdiction, e.g. national (exclusive economic zones, EEZs) or international fisheries, whether in two EEZs (on shared stocks), in an EEZ and the high seas (on straddling stocks), or at regional level (e.g. tuna or Antarctic fisheries);
— location in the production chain (capture, processing and distribution);
— supporting activities (maintenance, provisioning, etc.); and
— landing base, e.g. rural versus urban fisheries.

Though valid across a wide range of cases, these basic typologies may disguise more complex features. For instance, bottom fish could be of low value and some pelagic fish fetch record-high prices (e.g. bluefin tuna). Some species have bottom and pelagic characteristics (e.g. Alaska pollock). Some fisheries may be both small- and large-scale (e.g. large freezer mother boats using contracted canoes as fishing units). Small-scale fisheries may be technologically sophisticated and highly productive^1^ and a growing number of them export their production.

#### (i) Fleet size distribution

There are no complete or consistent time series but according to FAO (2009, SOFIA 1990–2008), the global fleet size, all vessel sizes included, had doubled from about two million vessels in the 1970s to some four million in the 2000s. The largest number operates from Asia. According to FAO (2009), the size of the Chinese fleet of vessels over 100 tonnes in 1996 was approximately 15 000. Adding these to the vessels registered by the Lloyds Maritime Information Services (LMIS; FAO 1999, p. 73) leads to an estimate of the world fleet size of 43–45 000 vessels over 100 tonnes in 1996. No data have been found about its evolution since then, but FAO (2009, fig. 18) indicates that the world fleet size as now registered in the Lloyds database has remained practically identical in number and tonnage. About 500 new industrial vessels were built every year in the 1950s, growing to about 2000 per year in the mid-1970s, and decreasing rapidly to about 300 per year in the early 2000s (Garcia & Grainger 2005)^2^ and to 50 vessels per year in 2007 (FAO 2009, fig. 19). Recent data seem to confirm that the period of large investment in large-size vessels, which peaked around the mid-1980s (Garcia & Grainger 2005), is largely over. However, the global fleet capacity index (fishing power) appears to have increased by a factor of six between 1970 and 2005, a period during which the global harvesting productivity decreased by the same amount (World Bank 2009).

### (d) Contribution to food security

Food security is achieved when ‘all people, at all times, have physical, social and economic access to sufficient, safe and nutritious food to meet their dietary needs and food preferences for an active and healthy life’ (FSN Forum 2007). Fishes have always been an important component of human food, particularly around lakes, rivers, deltas, floodplains and coastal areas, and particularly on small islands. This importance has spread globally with the development of trade. Fisheries may contribute to food security in two ways: (i) directly as a source of essential nutrients; (ii) indirectly as a source of income to buy food. Because of their contribution to total global output, and to the numbers of people involved in fishing, marine capture fisheries play a substantial role in these respects.

#### (i) Fish as food

**Figure 5.**
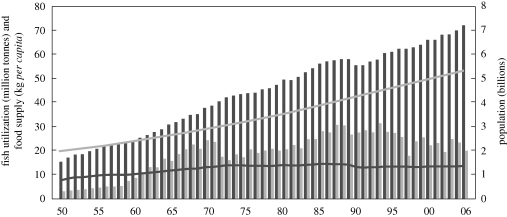
World fish utilization and supply. Dark grey bars, food; light grey bars, non-food uses; light grey line, population; dark grey line, food supply. Source: FAO (2009).

Fish is highly nutritious, rich in essential micronutrients, minerals, essential fatty acids and proteins, and represents an excellent supplement to nutritionally deficient cereal-based diets. It provides more than 1.5 billion people, particularly in low-income food-deficit countries, with almost 20 per cent^3^ of their average *per capita* intake of animal protein (FAO 2009). According to Worldfish, 400 million poor people depend critically on fish for their food,^4^ particularly in small island states, Bangladesh, Ghana and in the lower Mekong basin (FAO 2007; Hortle 2007; Laurenti 2007). From the 144 million tonnes produced in 2006 by capture fisheries (53%) and aquaculture (47%), about 110 million tonnes were used for food directly and 33 million tonnes indirectly through fish meal used for aquaculture, cattle, pig and poultry farming. This represented a record level of *per capita* supply of 16.7 kg (13.6 kg excluding China and 13.8 kg in low-income food-deficit countries). Outside China, *per capita* supply has shown a modest growth rate of about 0.5 per cent per year since 1992. Since 1950, the increases in fishery production have managed to offset demographic growth, gradually improving food supply from aquatic resources (figure 5).

#### (ii) Fish as a source of livelihood

The fisheries and aquaculture sector contribution to gross domestic product (GDP) typically ranges from around 0.5 to 2.5 per cent, but may exceed 7 per cent in some countries, a level similar to agricultural sector GDP. Growth in sector employment, particularly in the developing world, and largely outpacing that of agriculture, has been mainly in small-scale fisheries and in aquaculture. Capture fisheries provide employment and income directly and indirectly, e.g. through boat building, equipment and maintenance, vessel supplies, fish processing and trade, etc. Some 42 million people work directly in the sector, the great majority in developing countries. Adding the related activities, the sector is estimated to support more than 500 million livelihoods (Worldfish 2009), much of which is associated with marine capture fisheries. Moreover, fishery trade (including the sale of fishing agreements) is particularly important as a source of foreign currency for many developing countries. The sector also has particular significance for small island states. However, fisheries can also incur substantial costs to society, in lost resource rent—estimated at around 50 billion USD according to the World Bank (2009)—and/or in direct subvention including capital support and fuel subsidies, amounting to tens of billions USD per year.

#### (iii) Food security and poverty

Poverty is one of the sources of fishery resources degradation in many rural areas of the developing world and is an obvious constraint to achieving food security. However, healthy fisheries may contribute to poverty reduction through generation of revenues and wealth creation, operating as a socio-economic ‘lift’ at community level and contributing to economic growth at national level. If well managed, fisheries can maintain a sustainable stream of economic benefits in the community, and in some cases can operate as a safety net when needed, e.g. for people displaced from their area by serious drought (e.g. collapsing the agriculture sector) or by civil wars. However, though regionally important, particularly in Asia, fisheries are for the most part a small socio-economic sector, and cannot alone counteract poverty processes. Thus, impacts on poverty will be complementary to other sectors' contributions in nationwide poverty-reduction programmes (Béné *et al*. 2007).

## 3. Main drivers and constraints

The dynamics of the fishery sector reflect the complex interaction of a number of internal or external drivers, the most significant of which are examined below.

### (a) Demography, globalization and economic development

World population is a key driver of seafood demand and fisheries development. The projected increases in global population also suggest continued migration to coastal areas with accompanying development pressures, and increasing gaps between wealthy and poorer nations and peoples. Half of the world population lives within 60 km of the ocean and three-quarters of the large cities are located by the coast. By 2020, it is projected that some 60 per cent of the world population (about 6 billion) will live in coastal areas (Kennish 2002, in UNEP 2007). By 2050 it is expected to reach 9 billion (UN-DESA 2009) and according to UN-Habitat (2009), globally, 70 per cent of this population will live in urban centres. Most of the megacities (over 20 million inhabitants) will be in the coastal zones, looking for food and livelihoods.

Demand for fish as food is particularly high in the wealthier parts of society and demand increases with the economic level of development and living standards. This demand has been rising in both the developed and developing world at more than 2.5 per cent per year (Peterson & Fronc 2007), and as wealth increases in highly populated countries such as China and India, demand levels are likely to rise more strongly.

The issue of globalization and its application to fisheries can be controversial and politically sensitive. According to Held & McGrew (2000, cited in Rood & Schechter 2007): ‘… globalisation denotes the expanding scale, growing magnitude, speeding up and deepening impact of interregional flows and patterns of social interaction. It refers to a shift or transformation in the scale of human social organisation that links regions and continents’. The geographical expansion of fisheries has progressively globalized its structure, operations, trade flows, science and governance, at an increasing pace. The expansion of fleets onto the high seas has had a significant international impact on policy (e.g. the 1995 UN Fish Stocks Agreement; FAO 2009 Port States Agreement) and on its scientific support. Globalizing markets have increased demand and enhanced competition, affecting the evolution of the production and consumption patterns of the sector as well as wealth distribution within the sector. The strengthening and harmonization of food safety regulations and norms have changed seafood processing standards globally and can represent significant additional costs for exporters, with particular impacts in developing countries.

The global marketplace effect on scarce/high value resources has also shifted seafood products away from poorer consumers to those with greater ability to pay, with potentially significant local food security consequences. Environmental awareness of consumers, stimulated by public and environmental group campaigns, has increased demands for seafood products meeting demands for both quality and environmental sensitivity (Peterson & Fronc 2007). Ecolabelling is slowly spreading (Phillips *et al*. 2003; Seafood Choices Alliance 2008), largely through non-governmental efforts (e.g. the Marine Stewardship Council, MSC), and is likely to continue, better linking the role of governments, responsible for establishing management systems and norms, with independent third-party certification mechanisms. The public sentiment for sustainably produced food and retailers responding to that demand, particularly in Europe and North America, has contributed to improving management frameworks for capture fisheries, as shown by a decade of experience in developed nations.

### (b) Governance

Fisheries governance is an intricate web of public, private and hybrid institutions interacting in a complex manner to administer and regulate the sector (Garcia 2009*a*,*b*), and its weakness is considered to be the main factor behind the problems of overfishing and stock decline (Beddington *et al*. 2007; Garcia 2009*a*,*b*; Mora *et al*. 2009). Fishery sector governance and the systems within which it is nested are key drivers of fisheries performance. The governance frameworks adopted at national, regional and global levels interact with each other in a continuous but asynchronous manner (i.e. developing at different speeds in different places). The most crucial aspects of fisheries governance relate *inter alia* to: (i) connecting the fishery policy framework within a supporting national policy framework; (ii) the capability of fishery administrations; (iii) the nature of entitlements to resource access, including possible co-management systems; (iv) the level of participation of stakeholders, non-governmental organizations (NGOs) and civil society groups; (v) the availability and enforcement of deterrence measures; (vi) the level and extent of inter-ministerial coordination; and (vii) the quality of international collaboration.

The central international law and policy framework, the 1982 United Nations Convention on the Law of the Sea (UNCLOS), came into force only in November 1994. In the wake of the UNCED (UN Convention on Environment and Development), the implementation framework of UNCLOS has started to improve in a number of directions, with the adoption of the 1993 FAO Compliance Agreement, the 1995 United Nations Fish Stock Agreement and the 1995 FAO Code of Conduct for Responsible Fisheries (CCRF). The Precautionary Approach to Fisheries (PAF) and the Ecosystem Approach to Fisheries (EAF) were adopted in 1995 and 2001, respectively. The Sustainable Livelihood Approach to Fisheries has also been successively tested, particularly on small-scale fisheries (Allison & Horemans 2006). New instruments have been developed to combat IUU fishing such as the 2001 FAO International Plan of Action to Prevent, Deter and Eliminate Illegal, Unreported and Unregulated Fishing (IPOA-IUU) and, very recently, the legally binding 2009 Agreement on Port State Measures to Prevent, Deter and Eliminate Illegal, Unreported and Unregulated (IUU) Fishing.

On the high seas, which produce around 10 per cent of the world catch, weak governance resulting from incomplete jurisdiction by Coastal States has been a major problem. This area is plagued by the insufficient exercise of their international responsibilities by Flag States, Coastal States and Port States. As a result, the Regional Fisheries Management Organisations (RFMOs) are still unable to fully control member states' fishing activities and are confronted with IUU fishing. In some RFMOs, the parties themselves are setting catches well above scientific advice and failing to implement strong enough conservation measures. The rapid development of new fisheries in particularly vulnerable areas such as the deep sea is also trying the RFMOs' capability. For example, seamount fisheries or new fisheries in the Arctic (as sea ice retreats) are not clearly in the purview of existing RFMOs—though they potentially could be.

In the EEZs, jurisdiction is either purely national, shared (for transboundary stocks^5^) or harmonized (for straddling stocks^6^). In addition to the dearth of bilateral sharing agreements, weak governance problems encountered are connected mainly to the lack of clear and defendable entitlements (whether communal or individual), the widespread reluctance to limit access to resources, and the difficulty to eliminate excess fishing capacity, with the hard socio-economic and political consequences that this entails. The ongoing shift to participative and adaptive methods such as the Ecosystem Approach to Fisheries (EAF) has the potential for broadening the range and role of stakeholders. However, a major problem worldwide is in the efficient and effective management of small-scale fisheries with its special prescriptions including management subsidiarity, active participation and devolution, communal rights, self-management capacity-building and the use of sustainable livelihood approaches.

While advances such as those noted have addressed loopholes in the UNCLOS regarding stocks located wholly or partly on the high seas,^7^ decisive progress has also been seen in EEZs (e.g. in the US, Canada, Iceland, Norway, Australia, Namibia, Chile, New Zealand), adopting a progressive consensus on rebuilding stocks by reducing capacity, limiting catch or effort and using various forms of fishing rights to strengthen conservation incentives in medium- and large-scale commercial fisheries. Initial progress is also being made in implementing the EAF in many national fisheries, testing tools and approaches. Demonstrable progress in some fishery management systems, and recovery of depleted resources (Rosenberg *et al*. 2006; Beddington *et al*. 2007; Garcia 2009*a*,*b*; Worm *et al*. 2009) provide signs of hope even though achievement of the 2015 World Summit on Sustainable Development goal is yet distant. The management of small-scale fisheries, with its fundamental components of demography, poverty and food security, remains particularly problematic.

The performance of the governance system is reflected in the state of the resource base, the economy of the sector and the contribution to food security. Regarding the resource base conservation, with a few notable exceptions, performance has been poor. Since its entry into force in 1994, the UNCLOS has been an important improving factor, though partly counteracted by IUU. Regarding the economy, the Great Law of Fishing of Graham (1935), according to which all unlimited fisheries were to decline, has been amply verified (e.g. Garcia & Newton 1997; World Bank 2009). The latter study confirmed that 75 per cent of the world's fishery resources were economically underperforming assets leading to a loss of potential net economic benefits from marine fisheries of about 50 billion USD annually. This confirms that despite substantial improvements in policy and management frameworks, implementation remains sluggish, slowed by delayed response of stocks (because of their inherent dynamics or climate conditions), lack of political will and implementation capacity, unclear or inexistent users' rights, poor incentive structures (including corruption), etc. In both the high seas and EEZs, the highly dynamic nature of fisheries stocks and activities can make it difficult for governance systems to adapt quickly enough, unless a protective precautionary approach is applied. However, this can also result in increased inefficiencies, loss of benefit and increased compliance problems. Various approaches to adaptive management are being promoted to improve dynamic response, but these are yet to be widely applied. Regarding food security, the sector has performed well globally, improving *per capita* seafood supply despite large population increases. However, there is clear evidence that global capture fisheries reached their production limit in the late 1980s and that, on average, the quality of supply has decreased (smaller individuals and species).

Collaboration has improved between international institutions in charge of fisheries (FAO, International Council for the Exploration of the Sea—ICES, RFMOs) and those dealing with the environment (such as United Nations Environment Programme (UNEP), Convention on Biological Diversity (CBD), International Union for Conservation of Nature (IUCN), Convention on International Trade in Endangered Species (CITES), OSPAR, etc.) and the role of NGOs has been increasing significantly. However, though environmental and fishery governance are co-evolving, better collaboration and more explicit allocation of responsibilities are needed. The arenas for testing such collaboration are in area-based integrated management, such as ecosystem-based fishery management (EBFM) or the EAF, using *inter alia* marine-protected areas, refugia and marine spatial planning (Ehler & Douvere 2009). In both governance systems, the role of the civil society has grown with participative governance, raising public attention to fisheries and to broader environmental problems and changing the political and economic forces at play. This change, already occurring in many regions, better reflects societal concerns and aims than the more insular sectoral focus of the past. Finally, the uncertainty resulting from the complexity of international and national governance conditions may call for the application of well-considered precautionary approaches (FAO 1996) and other environmental management strategies.

#### (i) Fishery management science

In the context of governance, the description and status definition of fisheries systems and the science underpinning their management have long been a practical, theoretical and organizational challenge. Fishery management science has also been affected by globalization in many contexts. To a great extent, the scientific approach in the developed world and for large-scale fisheries has moved towards ever more complex data and modelling approaches, incorporating information from highly developed monitoring programmes including research surveys, sophisticated statistical modelling approaches and projections of future states of the resource. In the developing world and in small-scale fisheries (most of which remain practically unmanaged), scientific support is slowly moving towards integrated multi-disciplinary and participative assessment, with strong social sciences input, for example in the framework of the Sustainable Livelihoods Approach (Allison & Horemans 2006). The development of methodologies to advice management in data-limited situations is a priority. Many conventional methods are data-intensive and difficult to use in less developed countries and in the face of climate change. Simpler, compelling advice is needed that can be developed rapidly as changes are observed, coupled with adaptive management processes that can react effectively when better information becomes available.

### (c) Fishing capacity and technological progress

As discussed in §2*c*, the large vessel fleet has stabilized in size if not in fishing power (FAO 2009; World Bank 2009). In EEZs, however, the total number and power of smaller boats has increased substantially during the same period. As a consequence, global fishing capacity is still very high, probably at its highest point ever and, with some notable exceptions, the required global adjustment to reduced stock productivity has not yet happened. However, with fishery resources severely depleted, oil prices increasing, and subsidies decreasing, further massive investments are much less likely. Under these conditions, and considering the low rate of recruitment of new vessels, the fleet should decrease in numbers in the future to about half the present size (Garcia & Grainger 2005). With the slow but accelerating adoption of fishing rights, the fleet reduction might accelerate. A risk exists, however, that ageing vessels, trying to reduce operations costs to remain profitable, may reflag and move into IUU fishing. Furthermore, there is some evidence that fleet size is still increasing in some developing countries (e.g. Vietnam) despite the challenge of rising fuel costs and declining fishery resources.

Technological progress has been both a source of beneficial expansion and wellbeing for fishing communities and a constant challenge for managers. Fishing power and efficiency has increased dramatically because of larger or more powerful engines capable of propelling larger vessels and a greater amount of gear over a greater range. Other innovation areas include hydraulic power applications; stronger materials for fishing gears increasing size and efficiency; better electronic aids for navigation, bottom mapping, fish finding, gear deployment and communication; and improved fish preservation technology. Many of these technologies have also become inexpensive and compact enough to be available to almost any size vessel. Technology has improved fishing capacity and efficiency as well as safety on board, and in some cases improved fishing selectivity and product quality, but it has also greatly increased fishing mortality, spreading overfishing worldwide (Garcia & Newton 1997). Its unbridled use will continue to direct fisheries on a trajectory of progressive automation and reduction of labour, with negative implications for coastal communities. In addition, the drive for processing-based value-addition can keep fleets in operation in otherwise unviable conditions, even though resources are driven down to dangerously low levels. The reduction of discards in the 1990s (Kelleher 2005), essentially through improved transformation of the bycatch into edible products and fish feeds (as opposed to improved selectivity), is a case in point.

The impact of progress in information and communication technology includes: (i) improved information on vessel distribution (through satellite vessel monitoring systems, VMS); (ii) accelerated submission of catch data (e.g. through the VMS or the Internet); (iii) facilitation of global or regional information systems (e.g. on resources or IUU) and comparable research programmes on similar ecosystems; and (iv) improved understanding on underwater habitat (e.g. with autonomous underwater vehicles and improved scanning instruments), species distribution and migration and related environmental conditions (through archival tagging). However, it has also increased communication, foresight, evasive capacity and efficiency of pirate fleets. Fuel efficiency has been also improved and fishing is globally more fuel-efficient than any terrestrial meat production system (Tyedmers *et al*. 2005) but more efforts will be needed in the face of rising fuel costs. It should also be noted that although it is available, improved technology may not be necessarily applied unless both fishermen and government officials are willing to adopt it. This may require much greater incentives, particularly for technologies that improve reporting, monitoring and management capacity.

### (d) Climate change

Natural climatic oscillations, particularly those at medium (decadal) scale, have always affected fisheries as well as their management performance.^8^ Clearly, therefore, the impact of global climate change on ocean capture fisheries will be important for the availability, distribution and resilience of resources as well as for the sector structure and performance. Climate impacts are already evident, with warmer water species moving towards the poles, changes in coastal conditions that may affect habitat, impacts both positive and negative on productivity at all levels, and the effects of ocean acidification.

Climate change impacts will likely be as varied as the changes themselves and will be felt through changes in fishing opportunities (resources available and entitlements), operational costs (in production and marketing) and sales prices, with increased risks of damage or loss of infrastructure and housing. Fishery-dependent communities may also face increased vulnerability in terms of less stable livelihoods and loss of already insecure entitlements. Some changes may also be positive, opening new opportunities as new species become accessible. So far, most fishery sector literature concerns potential negative impacts and positive options are not well defined. A community's ability to limit losses and benefit from other opportunities will depend on its adaptive capacity. In terms of food security, climate change may potentially act across four interconnected dimensions: availability, stability, access and utilization of food supplies.

— Availability of fish will be affected by climate-driven changes in distribution. Fishers are very quick to find new opportunities and the overall long-term impact may be locally significant but globally minimal. In the transition period, however, large inter-annual fluctuations are to be expected, increasing uncertainty for fishers. Governance will need to detect and control fishing capacity developments fast enough to: (i) reduce stress on declining species; and (ii) let the new species settle successfully, avoiding overexploitation. Availability to consumers will depend on governance performance and eventual redirection of global trade flows and cannot be generically predicted.
— Stability of supply: changing conditions may lead to a period of quantitative and qualitative instability in supplies. Local fishing, markets and consumers could adapt, developing their existing opportunistic behaviour, particularly if trade flows remain functional. Instability, however, is not favourable to international export markets and some well-established opportunities may be hard to sustain.
— Access to food: in the absence of species-specific access regulations, there may be enough flexibility for fishers to adapt to new resource characteristics. Where such access rights have been established and resources change, more flexible governance may be needed, facilitating the modification and trading of rights and striking mutual access agreements with neighbouring countries (sharing resources). Industrial fisheries and heavy land-based infrastructures will be at higher risk of losing access to their historical supplies and building in flexibility will be advantageous. Access to consumers will be affected by global and local prices, trade flows and changes in their economic conditions.
— Utilization: changes in utilization (e.g. for food versus feeds) will depend on the nature of the change in the resource base. Unless coastward population flows and global development patterns are inverted, demand for human consumption should continue to grow and the use of fish as feeds (fishmeals and oils) should decline, with potential effects on other sectors such as aquaculture or poultry production.

Unless climate change factors lead to major losses of aquatic productivity, e.g. through food chain disruptions or damaging levels of acidification, the global consequences of climate change on the contribution of fish to food security might be minimal. However, local consequences could be rather serious, particularly on poor rural coastal areas, and would need to be further assessed using high-resolution models and scenario-development processes.

The vulnerability of fishers and fishing systems to climate change would be determined by three factors: their exposure to a specific change; their sensitivity to that change; and their ability to respond to impacts or take advantage of opportunities. Fisheries presently located in the high latitudes or at the interface between two neighbouring ecosystems (e.g. Senegal, Angola) or else in very shallow areas (estuaries, deltas, coral reefs) will be among the most exposed. Coastal communities in low-lying areas and small island states will be at high risk of floods and extreme weather conditions, requiring protective infrastructures, early warning systems, education and perhaps relocation. In these circumstances, priority assistance, including disaster relief, would have to be given to poor coastal fishing communities, so often neglected and disenfranchised.

The capacity to change is a real issue, particularly in highly vulnerable areas and fisheries. The status quo not being an option, adapting to climate change is a necessity, requiring preparation and means. If the change were slow, adaptation would be easier. Thus, if the rate of change was lower than the rate of depreciation of investments, the industry would adapt much more easily than if not the case, when high costs and economic collapse would be more likely, and special funds might be required for emergency intervention. However, the most imperative adaptations might be required in means and infrastructures (e.g. roads, electricity networks, early warning systems and other general infrastructures) that are beyond the fishery system itself but would influence its capacity to adapt.

## 4. Conclusions and outlook

In the next 40 years, the marine capture fishery sector will face its most critical challenges. In the past, fishers successfully overcame their fear of the unknown, risking their lives in one of the world's deadliest activities, reaching farther and deeper to bring food and expand their livelihoods. They now need to control and often reduce their harvesting capacity or, unless subsidized, face directly the consequences of not doing so. The potential for sustaining catches, food output and value at or near current levels, and supporting the nutrition and livelihoods of many hundreds of millions of dependent people, will rest critically on managing fisheries more responsibly.

### (a) Broad perspectives

The sector's future, whether in the high seas or EEZs, will be significantly conditioned by the capacity to address key inter-connecting elements of global or/and local relevance, including: (i) its present state and characteristics; (ii) its intrinsic capacity to adapt to multiple internal drivers, i.e. its resilience; (iii) external drivers affecting natural and human sub-systems of the ecosystems; and (iv) the constraints that may limit or jeopardize governance efforts. In the near and medium term, the sector will continue to face four main, possibly conflicting, challenges: (i) reducing excessive harvest to rebuild overexploited stocks and improve sectoral performance; (ii) reducing fisheries and aquaculture environmental impacts; (iii) matching the growing demand of an increasing world population; and (iv) adapting management and communities to the effects of climate change.

For the long term, perspectives on these major global resource systems are constrained by the capacity to predict the evolution of political and economic systems: access to and cost of energy, the control of land-based degradation and contamination, and climate variability and change. First and foremost, the future for marine fisheries will be conditioned by sectoral and national social, economic and environmental governance (Beddington *et al*. 2007; Garcia 2009*a*,*b*; World Bank 2009; Worm *et al*. 2009).

The challenge is a dual one. On the one hand, a large part of the society no longer accepts damage to natural, public trust resources such as the world's oceans and is calling for a change in use and consumption patterns. On the other hand, the potential impacts of climate change stand to shake all acquired positions and certainties. Resources will need to adapt their distribution and productivity at an unpredictable rate. Fisheries will need to adapt to weather, resource and market changes, and avoid undermining the adaptive capacity of the natural system on which they depend.

Drivers and constraints (including governance) will shape the external envelope of all the acceptable trajectories of the fishery system (i.e. its domain of ‘viability’ *sensu* Aubin, in Cury *et al*. 2005). Under such conditions, prediction is hazardous. In national comparisons, apparently similar situations may hide local differences in drivers, mechanisms or capacity to change. Similarly, climate change, international policy drivers (e.g. United Nations Convention on the Law of the Sea—UNCLOS, World Trade Organization—WTO, CITES) and consumer preferences are global but their impacts will vary regionally and locally.

While some similarities with other food production systems are to be expected (particularly for aquaculture), capture fisheries are fundamentally different in terms of their linkages and responses to change and in food security outcomes. Unlike most terrestrial animals, aquatic animal species are poikilothermic (cold-blooded) and changes in habitat temperatures will more rapidly and significantly influence metabolism, growth, reproduction and distribution, with stronger impact on fishing and aquaculture distribution and productivity. However, the interconnectedness of aquatic systems allows species to change distribution more easily as ecosystems shift, to remain in their zones of preference. Finally, the greater genetic diversity of marine animals compared with farmed animals also favours adaptation to new conditions. Therefore, the fishery sector requires special consideration to ensure that policy and management responses to climate change are effective.

To the extent that present trends in fishery ecosystem parameters (e.g. fleets, resources, environment, governance) and external drivers (e.g. demography, economic development and environmental policies, climate change) may provide some indications about the future, the following sections offer some reflections.

### (b) Fish supplies

Particularly for systems with low fuel demands for catching effort, capture fisheries, together with some forms of extensive aquaculture, have among the lowest ecological footprint for animal proteins. Replacing fisheries supplies with equivalent terrestrial sources or with intensive forms of aquaculture would significantly add to global resource demands, and would be a substantial ecological burden. However, without a substantial reduction of fishing capacity and explicit stock rebuilding plans, the prospect for building and sustaining resources is not good. It could be expected that underexploited stocks could produce more if fully developed and that overexploited, depleted and recovering stocks could produce more if properly managed. However, given complex ecosystem dynamics, this theoretical output cannot simply be added to the production of stocks that are presently fully exploited. Trade-offs will need to be made between more or less productive stocks as a matter of societal choice, as some may have to be accepted in a sub-optimal (but sustainable) state in order to optimize the production of others. The existence of predator–prey relations across the network of resources means that dynamic adjustments will naturally take place. A recovery of predator stocks will lead to increased predation on species that are also exploited for fishing. The combined sustainable yield may not be much higher than the current yield.

The productivity of different ecosystems has changed and will do so further owing to changing environmental conditions such as habitat loss or gain, climate change and non-native species introductions. Analysis of historical data indicates that many fisheries systems had much higher productivity in the past (e.g. Rosenberg *et al*. 2005), which may well not be recoverable due to fishery depletion as well as land use and ecosystem level changes.

There are, most likely, no major new resources to develop, except perhaps krill and oceanic squid and there may be ecological reasons for not overexploiting these stocks, which are key foods needed by large marine predators to recover from overfishing and adapt to climate change. A major unknown, subject so far to very limited consideration, is the impact of massive coastal degradation and global contamination of the ocean, the ultimate sink of human pollution.

Finally, climate change may improve the conditions for some resources and worsen them for others. The past has shown that unfavourable climatic conditions combined with excessive fishing pressure led to collapse. Inter-tropical resources may be heading towards particularly unfavourable combinations of conditions with high human demands, emigration of resources towards more temperate areas, and weak governance.

### (c) Economic, market and trade factors

Demographic trends point to population increase, though economic trends are much less certain, as are the social implications and political consequences. Uncertainties in these domains tend to shape future scenarios around three broad options: (i) status quo, with present trends maintained within their envelope; (ii) significant improvement in democracy and governance; and (iii) significant collapse in democracy and governance. The probability of each is unknown but the status quo is often considered as the most likely. The future of marine fisheries in these three contexts is likely to be: continued decline of the sector in scenario (i); substantial improvement in scenario (ii); and rapid collapse in scenario (iii) (Garcia & Grainger 2005).

The world population is increasing and notwithstanding the present financial crisis, economic growth is still expected in many countries and an increase in the demand for high-quality seafood can be expected. Though aquaculture may fill the gap to some extent, its ability to overcome its own constraints is not fully definable. Potentially increasing prices will provide additional incentive for fisheries and aquaculture investments, and in the absence of effective management, this would lead to stock collapse, reducing supplies at high societal costs and with a potentially severe backlash for the image of fisheries. Demography-driven demands for employment may also make full- or part-time fishing more attractive or even one of the few options available. In developed countries particularly demand may increase on coastal resources for tourism and recreation, including recreational fishing—the impact of which can be substantial (Coleman *et al*. 2004; Taylor *et al*. 2007).

The increase in demand for products and employment may lead to: (i) political pressure to slow rebuilding plans; (ii) greater incentives for IUU fishing; and (iii) increasing pressure on near-shore coastal resources by subsistence or low-income fisheries as well as on high-value products from more mechanized and industrialized fishing.

It can be expected that developing countries will continue past trends of directing a large and growing part of their primary resources to export trade, in search of hard currency. Parallels may be drawn with the rapid increase in use of land in Africa and Latin America by sovereign funds and agro-industrial groups to countries with high food demand such as China and India. In fisheries, the equivalent is the granting of fishing access agreements and the reflagging of fishing vessels under the national flag of developing countries owning large fishery resources. Without a major modification of the socio-economic perspectives in these countries and, for instance, the development of alternative sources of livelihood, the risk is that their fishery resources will remain under very high pressure and the contribution of fish, particularly to local food security, may decrease.

An important driving factor will be the World Trade Organization and other agents, with, for example, the EU rules preventing importation of fish from IUU operations, connecting trade and the environment. Together with progress in ecolabelling and sustainable seafood campaigns, these have potential, at least in wealthier countries, to reduce demand and hence economic drivers for poorly managed resources. This should stimulate better management. However, this is limited by the potential for catches which are rejected by wealthier more ‘ethically’ driven markets to be redirected to countries and populations with much lower buying power, e.g. through increased intra-regional trade.

The form under which fish is consumed may not be very relevant for future food security and safety, except perhaps with regard to contamination. The important unknown, globally, is the destination of lower-value fish now going to reduction for fish meals and oils. As global demand increases, particularly for poorer communities and developing countries, these resources will be under tension between three main destinations: (i) the present usage, from animal feeding and increasingly for aquaculture; (ii) direct food for humans; and (iii) the food of the stocks of predatory fish species (e.g. tuna, cod) it is commonly intended to rebuild, an often overlooked demand.

### (d) Governance

The policy objectives for the sector cannot be to produce much more than it produces today from wild stocks. The aim can only be to maintain and optimize production and profitability, in terms of catch composition (species, age and size), nutritional quality, fuel consumption and the ecological footprint. This implies maintaining, or recovering when relevant, resources and productive ecosystems and facilitating their adaptation to climate change. Governance frameworks have substantially improved, including those on the high seas, and good examples exist to demonstrate the effectiveness of the instruments at hand. The global political will of governments to implement them effectively and eliminate loopholes must still be demonstrated, however, and developing countries will continue to require assistance in that regard. The growing concern regarding environmental degradation generally will add pressure to better conserve fishery resources and their environment.

The adoption of fishing rights in commercial and large-scale fisheries bears the risk of concentrating resources in fewer hands, disenfranchising coastal communities. Their application to small-scale fisheries (in the form of communal rights or territorial use rights) will continue to be tested and the long-term outcome is at this stage unclear. Many of the approaches to improve fisheries management (e.g. fishing rights, participative/adaptive management) and sustain adequate levels of social equity require a democratic environment that may be yet to emerge in some countries. Ministries in charge of environment and the civil society active in this domain are also gaining influence and societal support, and the role of environmental agencies in fisheries management (and exploited ecosystems) will increase, with consequences that can only go towards reduced use rates, with impacts on food security that are yet to be assessed. At the same time, these agencies need to deal more effectively with the often irreversible environmental degradations and contamination from other human activities that affect fishery resources.

#### (i) Science

When attempting to represent the functioning of productive ecosystems in the next 50 years, scientists face numerous sources of uncertainty affecting the quality of advice. The use of methodologies such as the ecosystem approach has increased the amount of uncertainty to be addressed. However, in the future, uncertainties will be reduced and with the closer association of social sciences, the quality of advice under uncertainty has the potential to improve. However, uncertainty will not be eliminated (Mangel 2000), and ultimate management performance is likely to depend on the trade-off between precautionary protection and responsive adaptation to emerging limitations and opportunities.

The actions required for maintaining the contribution of capture fisheries to food security in the face of climate change are similar to those already applied, with two aggravating factors: (i) overfishing which reduces resilience to environmental change so that climate change adds urgency to the classical rebuilding/recovery issues; (ii) transition through a progressively changing context, perhaps with periods of acceleration, adding a destabilizing factor to an already complex governance equation.

Facing environmental change and the broad range of its impacts will require concerted and determined action by all main stakeholders, linking private sector, community and public sector agents, at national and regional levels. A wide range of measures can be considered for anticipation, mitigation or adaptation to climate change.

— Industry will need to adapt its technology to changing resources, reduce fossil fuel consumption and emission of greenhouse gases. Elimination of all excessive fishing capacity is a must. In addition, industry should contribute more to the maintenance of ecological services already threatened by climate change, e.g. developing more environment-friendly gears and practices. The public sector should: eliminate all harmful subsidies; provide economic and social incentives; improve proactivity and responsiveness in institutional and legal frameworks; improve the flexibility of management measures (e.g. flexible fishing rights and closed areas); implement the ecosystem approach to obtain the necessary cross-sectoral integrated response, and identify any appropriate precautionary actions; develop contingency plans and insurance schemes as well as monitoring and early warning systems; adopt a multidisciplinary and participative science for risk assessment, scenario development and performance assessments; develop education and information programmes, particularly in the small-scale sector where the most vulnerable fishery systems are; and strike bilateral and multilateral agreements to enhance the mobility of fishing. Vulnerability should be explicitly tracked and adaptive livelihood strategies introduced or enhanced, coupling risk management with development planning. Special efforts will be needed to reduce poverty as an effective way to reduce vulnerability.
— Civil society and public interest groups, including industry and environmental NGOs, have a central role to play in: articulating private and public sector action; assisting governments in programme implementation; raising awareness among the public, industry and the public sector; and raising financial resources to assist the poorest and hence most vulnerable strata of the sector, promoting the building of long-term capacity instead of reacting to emergencies; using the emerging crises to address long-standing problems, e.g. restructuring the rebuilt fleet to eliminate overcapacity; relocating the communities in safer sites; or developing new non-fishery job opportunities.

## Endnotes

1 A Senegalese canoe used to land 8 tonnes of Sardinella per day in the late 1970s, while an Icelandic high-tech small fishing boat with only three men on board caught 240 tonnes of cod in one month in 2008 (G. Valdimarsson, FAO, personal communication 2009).

2 These conclusions are based on a demographic analysis of the fleet age structure in the Lloyds database before the registration of the Chinese fleet and need to be revised.

3 Probably underestimated in view of the under-recorded contribution of small-scale and subsistence fisheries.

4 See http://www.worldfishcenter.org/wfcms/HQ/article.aspx?ID=684.

5 Stocks that occur or migrate across national EEZ boundaries.

6 Stocks of fish that migrate between, or occur in both, the EEZ of one or more states and the high seas.

7 In IUU, illegal fishing also includes poaching inside EEZs.

8 This section draws extensively from FAO 2008.
